# Musculoskeletal ultrasound diagnosis of quadrilateral space syndrome: A case report

**DOI:** 10.1097/MD.0000000000024976

**Published:** 2021-03-12

**Authors:** Jingfeng Zhang, Tian Zhang, RuiHua Wang, Ting Wang

**Affiliations:** Department of Ultrasound, Baoji High-tech Hospital, Baoji, Shaanxi, China.

**Keywords:** musculoskeletal ultrasound, quadrilateral space syndrome

## Abstract

**Introduction:**

Quadrilateral space syndrome (QSS) is a peripheral nerve entrapment disease, which can be misdiagnosed in clinic. In the past, QSS was mainly diagnosed by clinical symptoms combined with magnetic resonance imaging (MRI), electromyography (EMG), and arterial angiography. There are few reports on the diagnosis of QSS by musculoskeletal ultrasound (MSKUS) combined with clinical symptoms.

**Patient concerns:**

A middle-aged female patient had posterolateral pain and numbness in her right shoulder for 2 months.

**Diagnoses:**

At first, she was diagnosed as suprascapular nerve entrapment, while EMG of suprascapular nerve and axillary nerve indicated that nerve conduction was normal. Then, MRI was performed, showing the shoulder had no abnormalities, and EMG and arterial angiography of upper limb showed no abnormalities too. Finally, she was diagnosed as QSS according to MSKUS and lidocaine block test.

**Interventions:**

Two sealing treatments of axillary nerve block in quadrilateral space under the guidance of MSKUS were performed.

**Outcomes:**

After 2 treatments, the pain and numbness in her shoulder disappeared, and her shoulder could move normally. There was no recurrence after 3 months of follow-up.

**Conclusion:**

MSKUS is an effective method to diagnose QSS. It is fast, convenient and inexpensive, and is worth popularizing in clinic.

## 1 Introduction

Quadrilateral space is a quadrilateral space composed of joints, muscles, and bones. It is structured with shoulder joint and teres minor as the upper boundary, teres major as the lower boundary, triceps brachii as inner boundary, and the surgical neck of the humerus as outer boundary. Posterior humeral circumflex artery (PCA) and axillary nerve pass through the QSS. When PCA and axillary nerve are compressed by the surrounding tissues, it causes a series of symptoms and signs called QSS.^[1]^ QSS is mainly diagnosed by EMG and clinical manifestations.^[2]^ Imaging diagnosis of QSS mainly includes MRI and arteriography of upper limb. In addition, MSKUS is a simple, inexpensive imaging modality and has high resolution to soft tissues, and can be used to diagnose QSS. In this case, we report a patient with QSS diagnosed by MSKUS. In this patient, normal EMG and imaging examination showed no abnormities. Finally, the disease was confirmed as QSS because lidocaine block test of axillary nerve in quadrilateral space showed positive result.^[3]^

## 2 Ethics

This report was approved by the Ethics Committee of Baoji Hi-tech People's Hospital, and written informed consent for publication was obtained from the patient.

## 3 Case report

**Figure 1:**
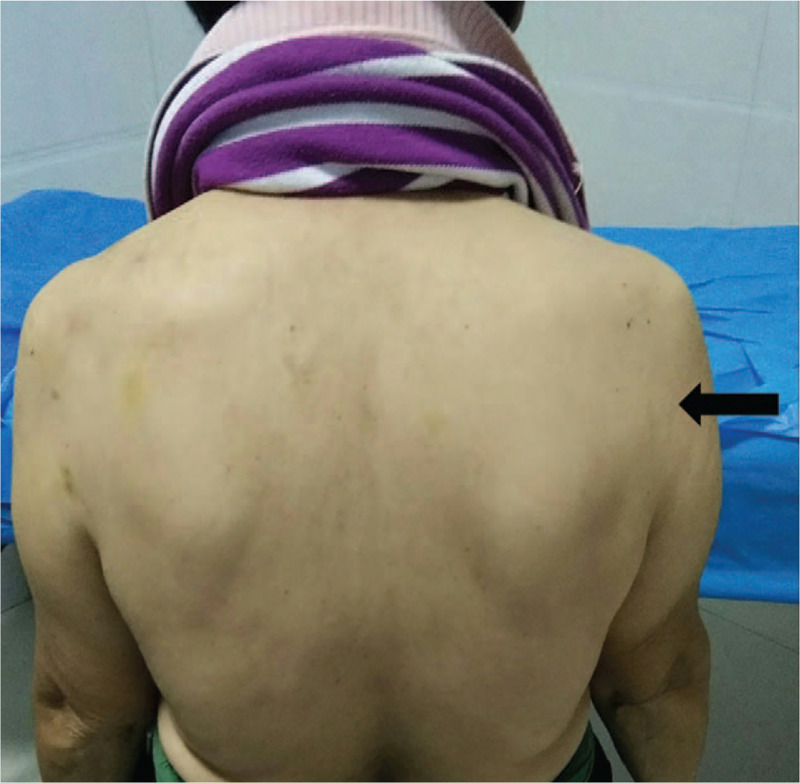
Pain in the right shoulder indicated by arrow.

A 50-year-old female patient, who was a sales person, had fatigue and weakness in the right shoulder and the posterolateral upper arm for 3 months (Fig. 1), with occasional pain. Recently, the pain was aggravated at night with intermittent numbness. The patient gave no history of trauma. On physical examination, there were tender points in the right shoulder and quadrilateral space behind the upper arm. The symptoms were aggravated when the shoulder joint was abducted and rotated externally.

**Figure 2:**
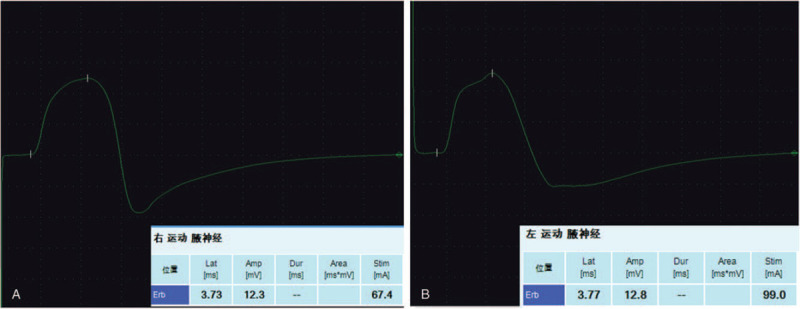
There was no significant difference in EMG between left and right sides, and both were normal.

**Figure 3:**
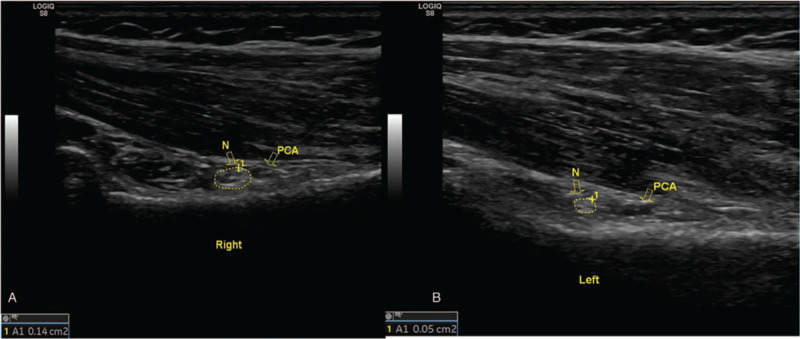
The cross-sectional areas of axillary nerves in the right and left shoulder were 0.14 and 0.05 cm^2^, respectively, and the right PCA was flat. N = axillary nerves, PCA = posterior humeral circumflex artery, Tm = Teres minor.

**Figure 4:**
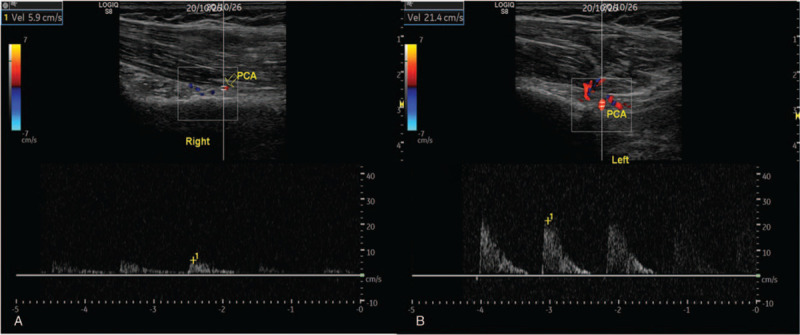
The blood flow velocities of PCA in the right and left shoulder were 5.9 and 21.4 cm/s, respectively. PCA = posterior humeral circumflex artery.

EMG of bilateral axillary nerve was performed in outpatient clinic, and it was observed that the latent time and conduction velocity of bilateral axillary nerve were normal, with no significant difference (Fig. 2). MRI of the right shoulder was normal, and arterial angiography of the right upper limb showed no abnormities. GE LOGIQ S8 diasonograph was used for MSKUS, and the broadband probe frequency was 5 to 11 MHz. MSKUS showed the ratio of longitudinal and transverse diameter of the right PHCA at the right quadrilateral space was 0.52, the blood flow velocity of PCA was 10.6 cm/s, the cross-sectional area of the axillary nerve was 0.14 cm^2^, the thickness of the deltoid muscle was 1.9 cm, and the thickness of the teres minor was 0.9 cm. The ratio of longitudinal and transverse diameter of the left PCA was 0.95, the blood flow velocity of PCA was 21.4 cm/s, the cross-sectional area of the axillary nerve was 0.05 cm^2^, the thickness of the deltoid muscle was 1.9 cm, and the thickness of the teres minor was 0.9 cm (see Figs. 3 and 4). The results of bilateral comparison showed that the longitudinal and transverse diameter of PCA at the right quadrilateral space decreased significantly, the blood flow velocity was slow, the axillary nerve was thickened, and the cross-sectional area was increased.

**Figure 5:**
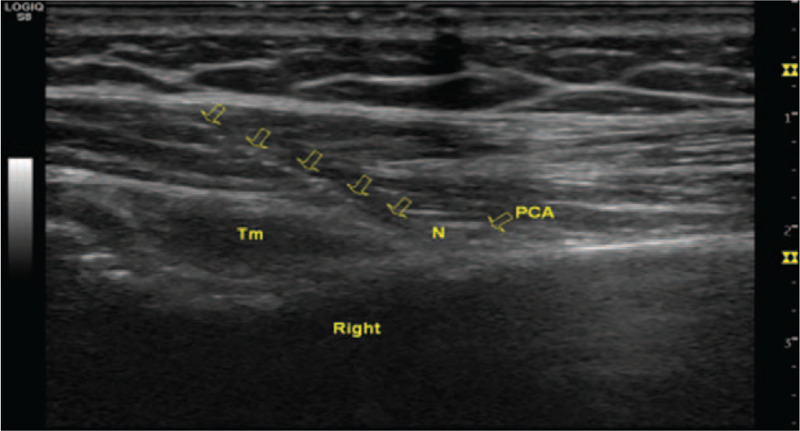
Axillary nerve block in quadrangular space under the guidance of ultrasound. N = axillary nerves, PCA = posterior humeral circumflex artery, Tm = Teres minor.

**Figure 6:**
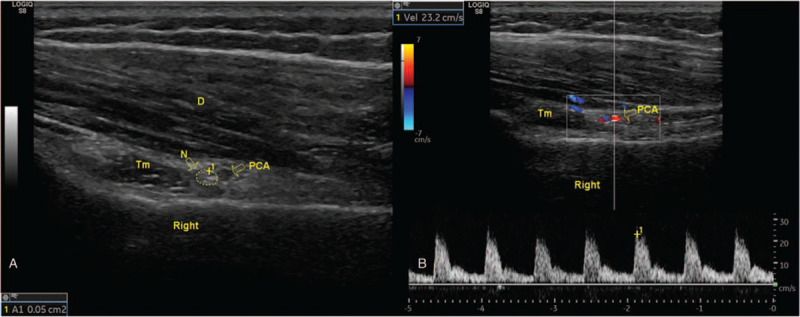
Three months after the treatment, the musculoskeletal ultrasound showed that the axillary nerve swelling disappeared, the cross-sectional area was 0.05 cm^2^ (A), and the blood flow velocity of the PCA returned to 23.2 cm/s (B). PCA = posterior humeral circumflex artery, Tm = Teres minor.

Treatment with axillary nerve block in quadrilateral space under the guidance of MSKUS was performed twice (Fig. 5). During follow-up, 1 month after operation, the symptoms disappeared. During follow-up, 3 months after operation, the symptoms disappeared; MSKUS showed that the ratio of longitudinal to transverse diameter of PCA increased from 0.52 to 0.97, the blood flow velocity increased from 10.6 to 23.2 cm/s, and the cross-sectional area of the axillary nerve improved from 0.14 to 0.05 cm^2^ (Fig. 6).

## 4 Discussion

QSS was observed and described by Cahinn and Palmer in 1983 for the first time.^[4]^ They described that axillary nerve and PCA are easily compressed in the quadrilateral space behind the shoulder. This disease is common in young people, especially in baseball or volleyball players, and is easily confused with frozen shoulder and rotator cuff injuries in clinic. The common causes include a history of shoulder trauma, tumors of humerus, tumors of surrounding soft tissue, glenoid lip cyst, thickened and edematous fibrous band, muscle enlargement.^[5–12]^ QSS sometimes have less typical clinical symptoms, making the diagnosis of the disease more difficult,^[13]^ so most of the previous reports applied imaging, angiography, and EMG techniques to diagnose the disease. When shoulder pain occurs, the preferred clinical imaging test is MRI, which can detect muscle atrophy around quadrilateral space, and helps exclude shoulder tumors and occupying lesions. Arteriography of upper limb is also of high diagnostic value for the diagnosis of pressure in PCA. Although it had been reported in the literature that the specificity of QSS diagnosis by arterial angiography arteriography is relatively low,^[14,15]^ EMG can detect latency and amplitude of axillary nerve to determine the degree of nerve damage, and can detect denervation potential of the muscles around the quadrilateral space.^[16,17]^ However, in a study of EMG detection of axillary nerve in QSS showed high false-negative rates.^[18]^ MSKUS can diagnose the pressure of PCA and axillary nerve in the quadrilateral space, and can also measure the difference in blood flow velocity of PCA between the affected shoulder and the unaffected shoulder.^[19–21]^ After comparison between the affected shoulder and the unaffected shoulder by MSKUS, we found that the cross-sectional area of the axillary nerves inside the quadrangular space of the affected shoulder was significantly increased and thickened; PCA became flat and the ratio of longitudinal and transverse diameter significantly decreased; the blood flow velocity of PCA slowed down significantly. The common accepted golden standard for the diagnosis of QSS is lidocaine block test. After injecting 5 mL of lidocaine (1%) into quadrangular space, the symptoms are obviously improved, which is seen as positive test.^[22]^ We also carried out such a test to establish the diagnosis of QSS. We performed treatment with QSS block in quadrilateral space twice under the guidance of MSKUS, and the patient's symptoms disappeared. Three months later, reexamination of MSKUS showed that the cross-sectional area of axillary nerve, ratio of longitudinal and transverse diameter, and blood flow velocity of PCA became normal, and there was no significant difference between the two shoulders.

This case is a 50-year-old middle-aged female patient, not an athlete and no history of trauma. No thrombus or obvious pressure was found in arteriography of upper limb. No atrophy of deltoid muscle and teres minor or lumps were found through MRI and physical examination. We speculate that although the patient was not an athlete, she worked as a saleswoman in a supermarket, which required abduction and lifting of her arms for a long time. This can cause axillary nerves to impinge frequently with the muscles around the quadrangular space leading to chronic injury. The repeated movement of the shoulder joint could make muscles around the quadrangular space inflamed and edematous, leading to pressure on the nerves and arteries and subsequent neuritis symptoms^[23]^ and pain. In this case, although PCA was compressed, the course of disease was short, which could be due to only mild pressure on the nerve and artery and short duration of muscle ischemia, with no atrophy of deltoid muscle and teres minor. No abnormities were observed in MRI and arteriography. The normal EMG report in this patient can be considered as a false-negative. In this case, MSKUS showed that PCA in the quadrilateral space was compressed and flattened. The blood flow was slow, and the axillary nerve was edematous and thickened, when comparing the 2 shoulders. As no abnormality was observed in other examination, and the duration of disease was short in this case, we speculate that the quadrilateral space was not compressed extensively.

## 5 Conclusion

To conclude, when a patient presents with pain and numbness in the back of the shoulder, the possibility of QSS cannot be excluded irrespective of the patient's age and occupation. In addition, if there are no abnormities in MRI, arteriography, and EMG, it is advisable to perform MSKUS and compare the results in both the shoulders to ascertain the diagnosis.

## Author contributions

**Conceptualization:** Jingfeng Zhang, Tian Zhang.

**Formal analysis:** JingFeng Zhang.

**Investigation:** Tian Zhang, Ting Wang.

**Writing – original draft:** Jingfeng Zhang, RuiHua Wang.

**Writing – review & editing:** Jingfeng Zhang, Ting Wang.
